# A case report of ABPA, cystic fibrosis and asthma treated with omalizumab

**DOI:** 10.1186/1939-4551-8-S1-A86

**Published:** 2015-04-08

**Authors:** JA Mazza, J Lewis, Tracy Gooyers

**Affiliations:** 1 St. Joseph’s Health Centre, Allergy Clinic, Toronto, Canada; 2 St. Joseph’s Health Centre, Respirology Clinic, Toronto, Canada; 3 Adult Cystic Fibrosis Program, Canada

## Introduction

In Canada the use of Omalizumab is restricted to patients with moderate to severe allergic asthma. However there are some cases reports showing its effectiveness in other conditions like chronic urticaria, ABPA and patients with nasal polyps and asthma. Although there are also reports of using omalizumab in patients with cystic fibrosis and ABPA, there are not controlled trials confirming their efficacy.

## Case report

A seventeen year old girl diagnosed with Cystic Fibrosis at the age of 6 and subsequently developed ABPA at the age of 15 but also had clear evidence of bronchial asthma confirmed by history and spirometry. She also had significant environmental allergies.

The diagnosis of ABPA was confirmed based on 5 or more criteria accepted by international committees and Cystic Fibrosis by genetic testing. She also had pancreatic insufficiency consistent with Cystic Fibrosis.

Because her condition was gradually deteriorating requiring despite the use frequent courses of antibiotics, prednisone and admissions to the hospital, it was decided to place her on omalizumab in August 2012. The dose based on her weight and total IgE was 225 mg every 2 weeks.

## Results

She felt better subjectively within two months after she was placed on omalizumab and after almost 2 years of treatment she had a significant improvement in all objective parameters including lab investigations, decreased need for steroids, antibiotics, admissions to the hospital and quality of life. She has not had any adverse reactions.

## Summary

It is known that Cystic Fibrosis together with ABPA will induce a more significant deterioration of the lung function than either alone and seriously interferes with quality of life. However, it appears that adding omalizumab to her current treatment has had an overall significant benefit that was sustained over 2 years.

The authors are aware that although it is unlikely that there will be RCT in this unique group of patients, case reports such as the one presented, might encourage others to follow the same approach in specific patients.

## Consent

Written informed consent was obtained from the patient for publication of this abstract and any accompanying images. A copy of the written consent is available for review by the Editor of this journal.

